# Virtual reality and pain management: The need for clarity for future interventions

**DOI:** 10.1177/20494637251319500

**Published:** 2025-03-16

**Authors:** Peter Snow

**Affiliations:** Institute of Orthopaedics and Musculoskeletal Science, Royal National Orthopaedic Hospital, 61142 University College London, London, UK

The prevalence of chronic pain in the United Kingdom and United States is 20–34%.^1,2^ High impact chronic pain (HICP) affects between 5.7% and 8% of adults in these countries alone^3,4^ and is associated with greater complexity as pain is more severe and there is a higher level of physical and mental health comorbidities.^5^ People with HICP require tailored intervention and a multidisciplinary approach.^6^ Therefore, a trend to supplement existing pain management has emerged, looking to technology as one possible solution, in particular to reduce treatment costs.

In the current issue of the British Journal of Pain, Tsigarides et al. examine the feasibility of using virtual reality (VR) in the treatment of fibromyalgia.^7^ Some of the earliest work on using VR for pain management started at the turn of the 21st century with Hoffman’s groundbreaking research on applying VR to pain management in people with burns.^8^ Other noteworthy initial work was conducted by Sander Wint and colleagues in 2002 using VR in a cohort of adolescents with cancer who needed lumbar punctures.^9^ Related to phantom limb pain management, Murray and colleagues in 2009 reported their work in using a VR headset paired with a data glove to replicate mirror box therapy virtually.^10^ These initial pioneers in VR for pain management used VR glasses or early headsets that we would now think of primitive, albeit expensive, technology. The biggest impact with regard to acceptance and widespread use of VR has involved two elements: firstly the proliferation and integration of High-Definition Multimedia Interface (HDMI) in the late 2000s and early 2010s, and secondly the Oculus Rift, first introduced as ‘Development Kit 1’ (DK1) in 2013. VR glasses and headsets had been brought to the consumer market before the DK1, most notably Sony’s HMZ-T1 headset in 2011, but with (now primitive) head-tracking, clarity, considerably lower price, and simple connectivity, Oculus Rift and the DK1 helped spur a new wave of VR for pain management research and its further acceptance in clinical practice.

Currently, what is missing in most studies that have applied VR to pain management is additional sensory feedback such as haptic, sound, or touch, for example. In their work on phantom limb pain management, Erlenwein et al.^11^ highlighted that sensory feedback would enhance VR treatments for this condition. In this context, we should consider how we define VR interventions as ‘immersive’ or ‘non-immersive’.

Force feedback, delivered through a haptic robot, is a technology that simulates tactile sensations by applying physical forces to the user. It enables a person to feel resistance, texture, or motion as if interacting with a real object. Haptic robots use sensors and actuators to measure the user’s input and provide corresponding force output, creating a realistic and interactive experience. This technology has been widely used in stroke therapy^12–14^ and surgical simulations to aid training utilising the sense of touch.

There are hundreds of studies now that have used a VR headset^15^ but, since 2013^16,17^ systems have been developed and clinical feasibility studies conducted to utilise not only VR headsets but also additional technology that allows haptic feedback such as force feedback in the treatment of phantom limb pain.^12,13^ With this move towards adding more sensory feedback into VR rehabilitation, now is an optimal time to re-examine our understanding and definition of immersive VR.

A further core consideration is the ever-evolving definition of virtual reality. Since the 1990s, VR experts have commented that the precise definition of virtual reality is vague.^18^ Original definitions were based on software representing three dimensional objects graphically.^17^ The most recent definition in the Oxford English Dictionary includes external hardware^19^ as technology has evolved. As a result, the lines are blurred when presenting studies which use VR for pain management: a wide range of methodologies have been used including studies that do not use a VR headset,^20–22^ those that use just a VR headset,^23,24^ and studies that utilise both a VR headset and additional sensory feedback.^16,17,25^ All this, before we consider more recent extensions to VR, such as mixed reality and augmented reality, collectively grouped as extended reality.^26^

One suggestion to streamline technical groupings is that interventions which use multiple sensory feedback in addition to a VR headset such as force feedback from a haptic robot, touch, enhanced sound, or even smell could be classified as ‘immersive’ VR. With purely VR headset interventions classified as ‘non-immersive’ VR.

With ever increasing numbers of studies being submitted and published using VR and different forms of extended reality for pain management, and, as products for VR pain management are becoming more common, setting clear boundaries and definitions on what exactly constitutes as VR intervention for pain management should be discussed.

As technology continues to develop, immersive VR will include further, increasingly refined, enhancements in sensory feedback. This includes further force feedback during rehabilitation interactions provided by haptic robots or vibrating motors (to provide simpler force feedback) to muscle activity via electromyography to allow user input and beyond, far removed from a simple computer screen. Distinguishing between the technologies used, precisely what each component is, and potential benefits and unwanted effects is imperative. Clear classification of VR interventions is urgently needed for the benefit of patients, clinical professionals, and researchers in the field of pain management and beyond this.
